# The impact of fluid resuscitation via colon on patients with severe acute pancreatitis

**DOI:** 10.1038/s41598-021-92065-7

**Published:** 2021-06-14

**Authors:** Tongtian Ni, Ying Chen, Bing Zhao, Li Ma, Yi Yao, Erzhen Chen, Weijun Zhou, Enqiang Mao

**Affiliations:** Department of Emergency, Ruijin Hospital, Shanghai Jiao Tong University School of Medicine, No. 197, Ruijin er Road, Huangpu District, Shanghai, 200025 China

**Keywords:** Pancreatitis, Translational research

## Abstract

Severe acute pancreatitis (SAP) is a life-threatening disease. Fluid Resuscitation Via Colon (FRVC) may be a complementary therapy for early controlled fluid resuscitation. But its clinical application has not been reported. This study aims to explore the impact of FRVC on SAP. All SAP patients with the first onset within 72 h admitted to the hospital were included from January 2014 to December 2018 through electronic databases of Ruijin hospital and were divided into FRVC group (n = 103) and non-FRVC group (n = 78). The clinical differences before and after the therapy between the two groups were analyzed. Of the 181 patients included in the analysis, the FRVC group received more fluid volume and reached the endpoint of blood volume expansion ahead of the non-FRVC group. After the early fluid resuscitation, the inflammation indicators in the FRVC group were lower. The rate of mechanical ventilation and the incidence of hypernatremia also decreased significantly. Using pure water for FRVC was more helpful to reduce hypernatremia. However, Kaplan–Meier 90-day survival between the two groups showed no difference. These results suggest that the combination of FRVC might benefit SAP patients in the early stage of fluid resuscitation, but there is no difference between the prognosis of SAP patients and that of conventional fluid resuscitation. Further prospective study is needed to evaluate the effect of FRVC on SAP patients.

## Introduction

Severe acute pancreatitis (SAP), with the mortality rate as high as 20–40%, accounting for about 20% of acute pancreatitis (AP), manifested as organ failure caused by systemic inflammation^1,2^.

Intravenous fluid resuscitation (IVFR) is the main treatment for SAP in early stage and aggressive volume resuscitation has become the cornerstone of treatment^3^. In the initial 24 h of acute pancreatitis, pro-inflammatory cytokines induce a variety of physiological changes leading to hypoperfusion of the organs of the whole body^4^. Under-resuscitation of acute pancreatitis in early stage is associated with increased risk of necrosis and mortality^5^ while over-resuscitation is also associated with a poor outcome^6^. Therefore, the method of fluid resuscitation needs to be optimized.

In recent years, strategies of goal-directed therapy or controlled fluid resuscitation using various parameters to guide fluid administration and reduce the risk of MODS gradually rise^7,8^. But the evidence that these treatments can reduce organ failure and mortality is still insufficient^7^. Fluid resuscitation via colon (FRVC), as a derivative of retention enema, is found to absorb large amounts of liquid and stabilize hemodynamics in the early stages of SAP^9^. It is a measure that allows the body to actively regulate water absorption and hemodynamics through colonic aquaporin^10^. FRVC has been proved to be effective in animal experiments^11,12^. There are also reports on the use of drugs or traditional Chinese medicine via colon^13–16^. In China, FRVC for SAP patients is a common clinical practice in some hospitals. This treatment method is mentioned in China's pancreatitis guidelines^17^, but it has not been reported as a strategy for fluid resuscitation because there is no relevant clinical research.

Hence, this study was aimed to determine whether FRVC reduces the early inflammatory response and improves the prognosis of SAP, and to provide a reliable clinical basis for FRVC of SAP in early stage.

## Results

### Patients

**Figure 1:**
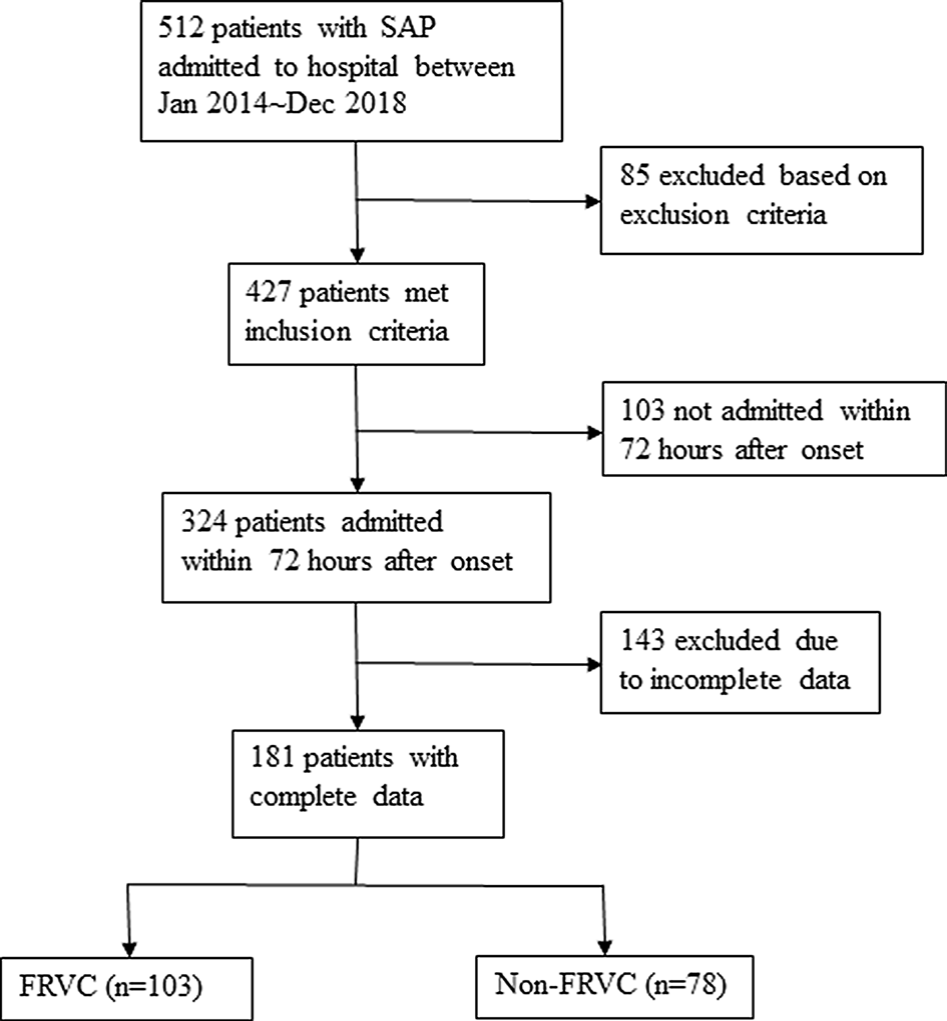
Flow chart describing the patients with severe acute pancreatitis between Jan 2014 ~ Dec 2018. SAP: severe acute pancreatitis, FRVC: fluid resuscitation via colon.

A total of 512 patients with severe acute pancreatitis aged from 17 to 89 years old were included, of which 85 patients were excluded based on exclusion criteria, 103 were excluded for not admitted within 72 h after onset and 143 were excluded due to incomplete data. The remaining 181 patients with a male-to-female ratio of 2.48:1 and a median age of 47.88 years were analyzed. Among them, 78 patients were treated with IVFR only. The other 103 patients were treated with FRVC and given intravenous fluid infusion at the same time (Fig. 1).

### Comparison of clinical and laboratory data between FRVC and non-FRVC group

There was no statistical difference between the two groups' clinical data (age, gender, body mass index, etiology of SAP, comorbidities), laboratory examination (white blood cell, CRP, PCT, hepatic and renal function and BISAP score). There was also no significant difference in 7-day and 28-day mortality and hospital stay between FRVC group and non-FRVC group (*P* > 0.05). The differences in baseline characteristics between FRVC and non-FRVC groups are described in Table 1.

**Table 1:** Baseline characteristics of patients with severe acute pancreatitis (n%; $\stackrel{-}{x}$±s).

	FRVC (n = 103)	Non-FRVC (n = 78)	*P* value
Mean age (years)	48.09 ± 14.54	47.60 ± 15.13	0.827
Gender (male%)	71 (68.93%)	58 (74.36%)	0.424
Body Mass Index (kg/m^2^)	25.27 ± 4.18	24.55 ± 4.59	0.275
**Etiology**			
Biliary	45 (43.69%)	38 (48.72%)	0.501
Hypertriglyceridemia	40 (38.83%)	28 (35.90%)	0.686
Alcohol	12 (11.65%)	7 (8.97%)	0.561
Others	6 (5.83%)	5 (6.41%)	0.870
**Comorbidities**			
Hypertension (%)	38 (36.89%)	31 (39.74%)	0.696
Diabetes mellitus (%)	22 (21.36%)	19 (24.36%)	0.633
**Indicators**			
White blood cell (× 10^9^/l)	12.89 ± 5.61	14.09 ± 6.18	0.174
C-reactive protein (µg/ml)	100.86 ± 80.90	105.43 ± 74.47	0.697
Procalcitonin (ng/ml)	5.39 ± 9.29	6.07 ± 11.73	0.670
Alanine aminotransferase (U/l)	65.50 ± 142.25	41.88 ± 42.39	0.114
Total bilirubin (µmol/l)	34.33 ± 41.23	38.30 ± 47.25	0.549
Blood urea nitrogen (mmol/l)	8.72 ± 8.29	8.99 ± 7.66	0.825
Serum creatinine (µmol/l)	136.28 ± 204.72	126.86 ± 164.92	0.738
BISAP score	2.61 ± 0.69	2.63 ± 0.65	0.870
Intra-abdominal hypertension (%)	44 (42.72%)	32 (41.03%)	0.819
Length of stay (d)	49.91 ± 41.16	50.81 ± 43.48	0.888
7-day mortality	1(0.97%)	3(3.85%)	0.316
28-day mortality	9 (8.74%)	5 (6.41%)	0.562

*BISAP score* Bedside Index for Severity in Acute Pancreatitis score, *BVE* blood volume expansion, *IVFR* intravenous fluid resuscitation, *FRVC* fluid resuscitation via colon.

*P < 0.05 was considered statistically significant.

### Comparison of fluid resuscitation between FRVC and non-FRVC group

The total amount of fluid received by patients in the FRVC group was greater than that in the non-FRVC group (*P* < 0.001), while the amount of fluid received through IVFR treatment was less than that in the non-FRVC group (*P* = 0.028). Significant differences were found in the time to reach the end point of blood volume expansion between the two groups (*P* = 0.030) (Table 2).

**Table 2:** **C**omparison of Fluid resuscitation between FRVC and non-FRVC group (n%; $\stackrel{-}{x}$±s).

Fluid resuscitation (BVE)	FRVC (n = 103)	Non-FRVC (n = 78)	*P* value
Total fluid (ml)	6932.83 ± 2955.83	5179.56 ± 2468.09	< 0.001*
IVFR (ml)	4355.15 ± 2474.25	5179.56 ± 2468.09	0.028*
FRVC (ml)	2597.09 ± 1291.11	–	–
Time of BVE (h)	24.42 ± 11.26	28.17 ± 11.65	0.030*

*BVE* blood volume expansion, *IVFR* intravenous fluid resuscitation, *FRVC* fluid resuscitation via colon.

*P < 0.05 was considered statistically significant.

### Comparison of inflammation indicators before and after treatment between FRVC and non-FRVC group

No matter in the FRVC group or the non-FRVC group, WBC decreased significantly after treatment compared with the baseline value (*P* < 0.001). However, the reduction of WBC in the FRVC group was more obvious (9.66 ± 5.24 vs. 12.10 ± 4.84 × 10^9^/l; *P* = 0.002). CRP was significantly reduced after treatment in the FRVC group (*P* < 0.001), while it was slightly increased in the non-FRVC group. Significant difference was found in CRP after therapy in the two groups (83.02 ± 65.28 vs. 106.59 ± 73.27 µg/ml; *P* = 0.024). PCT was also found to decrease after treatment, but it was only statistically significant in the FRVC group (*P* = 0.026) (Table 3).

**Table 3:** Comparison of inflammation indicators before and after treatment between FRVC and non-FRVC group ($\stackrel{-}{x}$±s).

Indicators	Group (n)	Before therapy	After therapy	*P* value
White blood cell (× 10^9^/l)	FRVC (103)	12.89 ± 5.61	9.66 ± 5.24	< 0.001*
	Non-FRVC (78)	14.09 ± 6.18 *P* = 0.174	12.10 ± 4.84 *P* = 0.002*	< 0.001*
C-reactive protein (µg/ml)	FRVC (103)	100.86 ± 80.90	83.02 ± 65.28	< 0.001*
	Non-FRVC (78)	105.44 ± 74.47 *P* = 0.697	106.59 ± 73.27 *P* = 0.024*	0.813
Procalcitonin (ng/ml)	FRVC (103)	6.07 ± 11.73	3.98 ± 7.67	0.026*
	Non-FRVC (78)	5.39 ± 9.29 *P* = 0.670	4.82 ± 9.93 *P* = 0.518	0.432

*FRVC* fluid resuscitation via colon.

*P < 0.05 was considered statistically significant.

### Comparison of organ failure before and after treatment between FRVC and non-FRVC group

There was no significant difference in modified Marshall Score, respiratory failure including PaO_2_/FiO_2_, renal failure including creatinine, heart failure including systolic blood pressure between the FRVC and non-FRVC group before and after treatment (Table 4).

**Table 4:** Comparison of organ failure before and after treatment between FRVC and non-FRVC group (n%; $\stackrel{-}{x}$±s).

Organ failure	FRVC (n = 103)	Non-FRVC (n = 78)	*P* value
**Modified Marshall score**			
Before therapy	3.16 ± 1.17	3.09 ± 1.39	0.731
After therapy	2.98 ± 1.27	3.00 ± 1.30	0.920
**Respiratory**			
Before therapy	98 (95.15%)	72 (92.31%)	0.429
After therapy	100 (97.09%)	75 (96.15%)	0.728
**PaO**_**2**_**/FiO**_**2**_			
Before therapy	190.43 ± 62.20	200.99 ± 59.75	0.251
After therapy	213.92 ± 51.98	208.12 ± 50.34	0.452
**Renal**			
Before therapy	20 (19.42%)	15 (19.23%)	0.975
After therapy	21 (20.39%)	11 (14.10%)	0.272
**Serum creatinine (µmol/l)**			
Before therapy	136.28 ± 204.72	126.86 ± 164.92	0.738
After therapy	119.60 ± 143.43	116.92 ± 147.08	0.902
**Cardiovascular**			
Before therapy	8 (7.77%)	10 (12.82%)	0.261
After therapy	6 (5.83%)	9 (11.54%)	0.167
**Systolic blood pressure (mmHg)**			
Before therapy	129.47 ± 24.70	125.87 ± 26.81	0.351
After therapy	132.88 ± 25.11	130.62 ± 27.07	0.561

*IVFR* intravenous fluid resuscitation, *FRVC* fluid resuscitation via colon.

*P < 0.05 was considered statistically significant.

### Comparison of complications within 24 h after the end point of blood volume expansion

The incidence of hypernatremia and the rate of mechanical ventilation in the FRVC group were lower than those in the non-FRVC group within 24 h after the end point of blood volume expansion (*P* < 0.05). There was no difference in the incidence of IAH between the two groups (*P* > 0.05) (Table 5).

**Table 5:** Comparison of complications within 24 h after the end point of blood volume expansion (n%).

	FRVC (n = 103)	Non-FRVC (n = 78)	*P* value
Hypernatremia, n (%)	18 (17.48)	24 (30.77)	0.036*
IAH at the end of BVE, n (%)	51 (49.51)	38 (48.72)	0.915
Mechanical ventilation, n (%)	37 (35.92)	41 (52.56)	0.025*

*FRVC* fluid resuscitation via colon, *IAH* intra-abdominal hypertension.

*P < 0.05 was considered statistically significant.

### Analysis of the relationship between hypernatremia and fluid type used for FRVC

All the patients with SAP in FRVC group were further categorized into the following subtypes: pure water group (n = 25), Ringer’s lactate group (n = 49) and normal saline group (n = 29). After adjustment of age, gender, body mass index, etiology, hypertension, diabetes mellitus, white blood cell, C-reactive protein, procalcitonin, alanine aminotransferase, total bilirubin, blood urea nitrogen, serum creatinine, PaO_2_/FiO_2_, systolic blood pressure, intra-abdominal hypertension, BISAP score, total fluid, time of blood volume expansion, modified Marshall Score , binary logistic regression analysis revealed that patients used pure water for FRVC had 0.077 times more likely to develop hypernatremia (95% CI 0.007–0.853, *P* = 0.037) (Table 6).

**Table 6:** Analysis of the relationship between hypernatremia and fluid type used for FRVC.

	Adjusted OR (95%CI)	*P* adjusted
IVFR (n = 78)	1	
**Fluid type of FRVC**		
Pure water (n = 25)	0.050 (0.004–0.633)	0.021*
Ringer’s lactate (n = 49)	0.926 (0.293–2.921)	0.895
Normal saline (n = 29)	0.811 (0.206–3.186)	0.764

*P* adjusted: assessed by logistic regression; adjusted for age, gender, body mass index, etiology, hypertension, diabetes mellitus, white blood cell, C-reactive protein, procalcitonin, alanine aminotransferase, total bilirubin, blood urea nitrogen, serum creatinine, PaO_2_/FiO_2_, systolic blood pressure, intra-abdominal hypertension, modified Marshall score, BISAP score, total fluid, time of blood volume expansion.

*P < 0.05, significant difference.

### Comparison of pancreatic necrosis between FRVC group and non-FRVC group

Four patients (1 in FRVC group and 3 in non-FRVC group) failed to complete enhanced CT because they died within 7 days. There was no significant difference in pancreatic necrosis between FRVC group and non-FRVC group (*P* > 0.05) (Table7).

**Table 7:** Comparison of pancreatic necrosis between FRVC group and non-FRVC group (n%).

Area of pancreatic necrosis	FRVC (n = 102)	Non-FRVC (n = 75)	*P* value
< 30%	43 (42.16%)	28 (37.33%)	0.518
30–50%	37 (36.27%)	24 (32.00%)	0.586
> 50%	22 (21.57%)	23 (30.67%)	0.158

*FRVC* fluid resuscitation via colon.

*P* < 0.05 was considered statistically significant.

Analysis of the relationship between 90-day mortality and fluid type used for FRVC.

All the patients with SAP in FRVC group were also categorized into the following subtypes: pure water group (n = 25), Ringer’s lactate group (n = 49) and normal saline group (n = 29). After adjustment of age, gender, body mass index, etiology, hypertension, diabetes mellitus, white blood cell, C-reactive protein, procalcitonin, alanine aminotransferase, total bilirubin, blood urea nitrogen, serum creatinine, PaO_2_/FiO_2_, systolic blood pressure, intra-abdominal hypertension, modified Marshall score, BISAP score, total fluid, time of blood volume expansion, binary logistic regression analysis revealed that there was no significant correlation between the types of fluid resuscitation and 90-day mortality (Table 8).

**Table 8:** Analysis of the relationship between 90-day mortality and fluid type used for FRVC.

	Adjusted OR (95%CI)	*P* adjusted
IVFR (n = 78)	1	
**Fluid type of FRVC**		
Pure water (n = 25)	1.238 (0.085–17.979)	0.876
Ringer’s lactate (n = 49)	2.133 (0.210–21.712)	0.522
Normal saline (n = 29)	0.283 (0.025–3.209)	0.308

*P* adjusted: assessed by logistic regression; adjusted for age, gender, body mass index, etiology, hypertension, diabetes mellitus, white blood cell, C-reactive protein, procalcitonin, alanine aminotransferase, total bilirubin, blood urea nitrogen, serum creatinine, PaO2/FiO2, systolic blood pressure, intra-abdominal hypertension, modified Marshall score, BISAP score, total fluid, time of blood volume expansion. *P < 0.05, significant difference.

### Comparison of 90-day survival rate between FRVC group and non FRVC group

**Figure 2:**
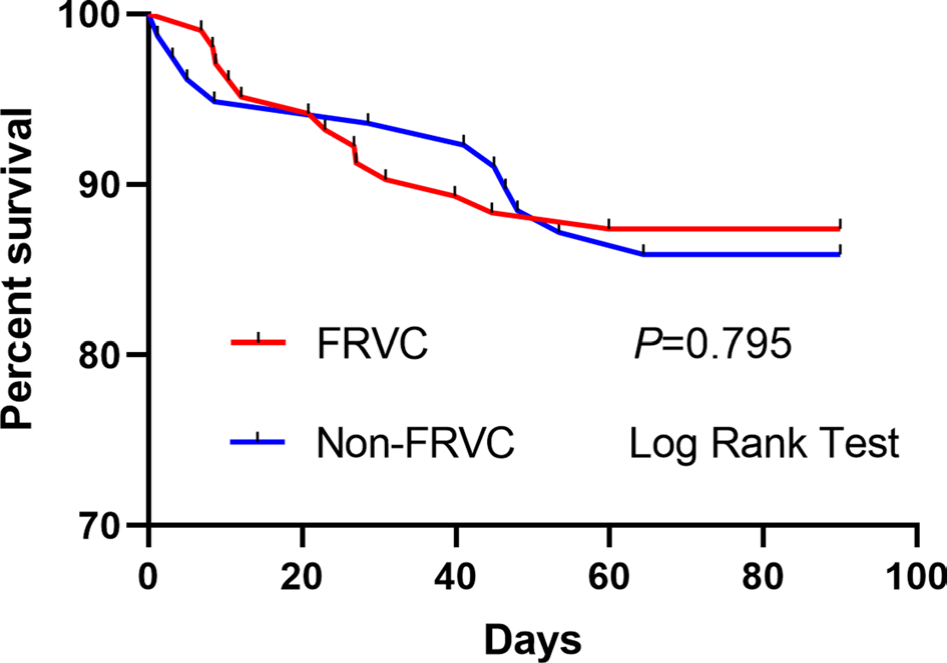
Kaplan–Meier 90-day survival between FRVC and non-FRVC groups in patients with severe acute pancreatitis. FRVC: fluid resuscitation via colon.

During the 90-day treatment, 90 (87.38%) of patients in the FRVC group were still alive compared with 67 (85.90%) of patients in the non-FRVC group. FRVC had no correlation with 90-day mortality (*P* > 0.05) (Fig. 2).

## Discussion

To our best knowledge, this is the first study to investigate the impact of FRVC on patients with SAP. How to reduce the early inflammatory response of SAP patients is currently a continuous problem during the entire fluid resuscitation period. Unfortunately, there is still no exact way to improve this problem so far. At present, the main role of fluid resuscitation is to maintain hemodynamics through the administration of fluids and electrolytes. In addition to intravenous routes, enteral routes can be used. The enteral routes relied on nasal feeding in the past. However, due to hemodynamic instability and nasal feeding intolerance in the early stage of SAP, the nasal feeding route has not been used for fluid resuscitation. So, based on the successful animal experiments^9^ and the experience of some Chinese clinicians in using FRVC, we tried to use FRVC as a supplement to IVFR.

In our study, the fluid volume of IVFR in the FRVC group was less than that in the non-FRVC group, but the overall fluid resuscitation volume was greater than that in the non-FRVC group. The results suggested that FRVC provided more liquid for aggressive fluid resuscitation. In SAP, increased capillary permeability leads to capillary leakage, which in turn leads to a significant decrease in blood volume^18^. The fluid sequestration level of SAP is higher than that of mild and moderately acute pancreatitis^19^. Therefore, the lack of blood volume in SAP is more serious and fatal. Study by Karin et al. in 2014 revealed that individualized optimization of fluid resuscitation in the early stages of SAP reduces vascular endothelial injury, pancreatic edema and inflammation^20^. The mechanism that the colon actively absorbs water through aquaporin^21^, slows down the absorption of fluid when the blood volume is gradually sufficient^10^. Because of the protective mechanism, more fluid can be put into FRVC without worrying too much about excessive fluid resuscitation. The present study showed significant difference between FRVC and non-FRVC groups in terms of shortening the time of blood volume expansion, suggesting that the speed of fluid resuscitation to reach the goal was significantly increased under the synergistic treatment of FRVC.

Inflammation indicators include WBC, CRP and PCT decreased significantly after FRVC compared to the baseline value. Among them, WBC and CRP declined more significantly in the FRVC group after therapy compared to the non-FRVC group, while the decrease in PCT was not statistically different between the two groups. The inflammatory response which causes multiple organ failure of SAP is related to prognosis^22^. As one of the evaluation indicators of SIRS, WBC is a predictive tool for the severity of acute pancreatitis^23,24^. CRP has some value in predicting SIRS or death in AP^25^. PCT has also been proven to have the value of predicting the severity and prognosis of SAP and can effectively support the guidance of antibiotic treatment^26^. It is also proved that the combined detection of PCT and CRP has a higher diagnostic value for judging the severity of pancreatitis^27^. Clinical study has shown that reducing the early inflammatory response of SAP patients may improve the condition of patients^28^. FRVC may have a better therapeutic effect on SAP by improving early inflammatory response.

Aggressive fluid resuscitation may cause respiratory complications, electrolyte metabolism disorders and IAH^29,30^. The rate of mechanical ventilation in the FRVC group were lower than those in the non-FRVC group. Respiratory failure usually occur in the early stage of SAP^31^. A meta-analysis of aggressive fluid resuscitation for acute pancreatitis involving a total of 2626 patients showed that patients receiving aggressive IVFR treatment are at higher risk of acute respiratory distress syndrome (ARDS)^29^. At present, the early prevention and treatment of respiratory failure mainly focus on the targeted treatment of risk factors, while other clinical treatment measures that can clearly alleviate ARDS are relatively few. A retrospective study shows that PCT and CRP are significantly increased in SAP patients with ARDS^32^. In our study, the two indicators decreased significantly after FRVC, indicating that FRVC reduced the proportion of mechanical ventilation in SAP patients. This provides us with a new way of thinking that FRVC can be used to reduce lung injury during early fluid resuscitation.

In our study, there was no significant difference in the proportion of pancreatic necrosis between FRVC group and non-FRVC group. Pancreatic necrosis is associated with mortality^33^. If infection occurs in necrotic tissue, the mortality rate of these patients is as high as 20–30%^34^. As early as 2002, study^35^ found that insufficient fluid resuscitation can lead to pancreatic necrosis. However, another systematic review^36^ reported that positive fluid resuscitation was not significantly associated with pancreatic necrosis. A multicenter retrospective study^37^ from Japan showed that there was no significant correlation between the amount of fluid given in the first 24 h and the incidence of pancreatic infection. These evidences suggest that pancreatic necrosis is no longer related to fluid volume, but may be related to the severity of the disease itself. In our study, FRVC group did not show the advantage in preventing pancreatic necrosis, which may be related to the positive fluid resuscitation in both groups.

Our previous study found that positive fluid resuscitation increased mortality and complications^38^. Aggressive fluid resuscitation in SAP patients was associated with increased incidence and longer duration of AKI^39^. The results showed that there was no significant difference between the two groups before and after treatment, and there was no significant difference in the failure of organs. However, the mechanical ventilation rate of FRVC group was low, but there was no significant difference between the two groups, which may be that patients in non-FRVC group increased the oxygenation index through the support of mechanical ventilation.

The incidence of hypernatremia in the FRVC group were lower than those in the non-FRVC group. There is still lack of direct evidence on how various types of fluids should be used for fluid resuscitation. In the current fluid resuscitation, crystal fluid is still the primary choice^40^. Research has shown that NS and RL are the most commonly used fluids in fluid resuscitation of acute pancreatitis^41^. The hypernatremia caused by early fluid resuscitation is related to the use of normal saline^42^. In this study, patients used pure water for FRVC had a significantly lower probability of getting hypernatremia. The possible reason is that the osmotic pressure of water is lower than NS or RL. Although the association between the incidence of hypernatremia and increased mortality has been confirmed in critically ill patients^43^, there is currently no sufficient evidence to show that actively correcting hypernatremia caused by fluid resuscitation in critically ill patients has a positive impact on clinical outcomes^44^. The results also found that there was no significant correlation between the type of fluid resuscitation in FRVC group and the 90-day mortality. Although studies have shown that the use of RL for fluid resuscitation in SAP patients has certain advantages^45,46^. A systematic study^47^ that included these two studies showed that sodium lactate Ringer's solution did not reduce mortality in patients with acute pancreatitis compared with normal saline. However, FRVC provides a potential measure to reduce blood sodium in early stage of fluid resuscitation.

In our study, 49.51% of patients in FRVC group had IAH at the end of BVE. The incidence was not significantly different from the non-FRVC group. Past researches show that the incidence of IAH is between 51 and 78%^48,49^. The amount of crystal fluid used in the initial resuscitation seems to be associated with the risk of IAH^50^, and IAH grade is an important predictor of mortality^51^. Compared with classic IVFR, FRVC did not significantly increase the incidence of IAH. This allows us to infuse more fluid into the colon.

In the current study, FRVC was not significantly correlated with 7-day, 28-day and 90-day survival and the overall survival rate of SAP is 87.38%. A retrospective cohort study shows that 357 (82.1%) patients survived the 90-day follow-up in a total of 435 SAP patients treated in the intensive care unit^52^. In some other studies, the 90-day mortality of SAP is between 11.9 and 15.1%^41,53^. This is consistent with our research results. The survival of SAP is related to many factors, including the severity of the disease, treatment methods and so on^52,54^. Mohamed et al.^29^ show that early aggressive IVFR does not improve the overall incidence of systemic inflammatory response syndrome, persistent organ failure, pancreatic necrosis, and mortality. Age, gender, heart disease, chronic liver failure and laparotomy affect the 90-day mortality^52^. Therefore, this study found that FRVC is a link in the entire SAP treatment process, but it does not play a decisive role in the prognosis.

## Conclusion

FRVC reduces the early inflammatory response of SAP patients and reaches the goal of BVE faster. Using pure water for FRVC can effectively reduce the incidence of hypernatremia. However, fluid resuscitation is only one aspect of SAP treatment while the pathophysiological state of SAP is constantly changing. Many factors affect the prognosis of SAP patients, especially under complex pathophysiological conditions. Though FRVC could not significantly improve the 90-day mortality rate, it provides a new way for fluid resuscitation and we encourage physicians to try to use FRVC as an auxiliary means for fluid resuscitation. Further prospective study is needed to evaluate the effect of FRVC on SAP patients.

### Limitations

This study had four major limitations. First, because it is a retrospective study, some data such as TNF-α and interleukin were missing and not included in the statistics. As our research is exploratory, sample was not estimated according to statistical power calculation. Second, speed, duration and fluid type used for FRVC were not consistent due to the lack of a uniform standard for FRVC. Third, the amount of fluid absorbed by the colon through FRVC is difficult to accurately count. This affects the calculation of total fluid resuscitation. Fourth, one reason to terminate FRVC was that the patient could not tolerate FRVC. This is a subjective feeling, and tolerance of each patients to FRVC vary from one another. It will eventually affect the execution of FRVC.

## Materials and methods

### Study subjects

Case review and data collection were through electronic databases of Ruijin hospital. The SAP patients aged > 16 with the first onset within 72 h admitted to the hospital were included in the present study from January 2014 to December 2018. Enrollment of SAP patients was based on Atlanta guideline^55^. SAP was regarded as any organ dysfunction of severity ≥ 2 lasting > 48 h according to the modified Marshall score^55^. The exclusion criteria were pregnant or lactating women; malignant tumors; chronic pancreatitis; severe chronic cardiovascular, kidney and liver diseases. This study was approved by Ruijin Hospital Ethics Committee affiliated to Shanghai Jiao Tong University School of Medicine and granted waiver of informed consent (2018241). Data analysis was performed in accordance with the principles expressed in the 1964 Helsinki Declaration and its later amendments.

Patients within 72 h of onset of pain were treated according to 2012 Atlanta guideline^55^ for the diagnosis and treatment. It includes fasting, gastrointestinal decompression, fluid resuscitation, maintenance of water and electrolyte balance, support of organ function, symptomatic treatment, intensive care and etiological treatment. All patients underwent goal-directed fluid resuscitation immediately after admission and were divided into two groups: FRVC group and non-FRVC group. The FRVC operation was to insert a disposable enema tube into the colon through the anus to a depth of about 25 cm, with the end connected to an infusion device. According to the tolerance of patients, intermittently infuse liquid into the colon at a rate of 250–500 ml per hour. Patients in the FRVC group received IVFR and FRVC at the same time. The type of fluid used for FRVC included sodium Ringer’s lactate solution (RL), normal saline and pure water. The condition for terminating FRVC was to achieve the goal of blood volume expansion (BVE) or the patient could not tolerate it. After the termination of FRVC, the patient continued IVFR treatment and was still included in the FRVC group. BVE is the first stage of fluid resuscitation. The endpoint of BVE was defined as two or more of the following requirements were met: heart rate < 120 beat per minute; mean arterial pressure 65 to 85 mmHg; urine output > 0.5 ml/kg/h; or hematocrit (HCT) 30% to 35%^8^.

### Clinical variables

The clinical variables were extracted from the electronic database of Ruijin hospital for each patient. Baseline demographic information including age, gender, etiology (biliary factors, alcohol, hyperlipidemia), biochemical indicators, systemic inflammatory factors (white blood cell (WBC), C-reactive protein (CRP), procalcitonin (PCT)), Bedside Index for Severity in Acute Pancreatitis (BISAP) score, were collected. CTSI was used to evaluate the degree of pancreatic necrosis within 28 days after admission^56^. Organ failure was evaluated according to the modified Marshall Scoring system^55^. Method of fluid resuscitation, type and dosage of fluid used for FRVC, time of blood volume expansion (TBVE), hypernatremia (defined as serum sodium greater than 145 μmol/L) within 24 h after the endpoint of BVE, mechanical ventilation within 24 h after the endpoint of BVE, incidence of intra-abdominal hypertension (IAH, defined as the intravesical pressure greater than 12 mmHg) on admission and at the end of FRVC. 7-day, 28-day and 90-day mortality were also collected. All patients discharged from the hospital within 90 days were contacted by telephone to investigate the 90-day survival rate.

### Statistical analysis

The clinical data were analyzed by SPSS 19.0 statistical software (SPSS, Inc., Chicago, IL). Chi-square test was used to compare categorical variables while Mann–Whitney U test was used to compare measurement data between FRVC and non-FRVC group. Paired t-test was used to compare inflammation indicators before and after treatment. Binary logistic regression was used to evaluate the relationship between the type of fluid used for FRVC and hypernatremia, and it was also used to evaluate the relationship between the type of fluid used for FRVC and 90-day mortality. The enumeration data were presented as the means ($\bar{x}$) ± standard deviation (SD). Categorical data were expressed as frequencies and percentage. P < 0.05 was considered statistically significant.

## Data Availability

The clinical data used for the analysis in the current study are available from the corresponding author on reasonable request in a de-identified manner.
